# Volumetric Analysis of Maxillary Sinus and Nasal Conchae According to Skeletal Classes and Cranio-Maxillary Relation

**DOI:** 10.3390/diagnostics15182319

**Published:** 2025-09-12

**Authors:** Berfu Çerçi Öngün, İbrahim Tekdemir, Seçil Aksoy, Nimet İlke Akçay, Kaan Orhan

**Affiliations:** 1 Department of Anatomy, School of Medicine, Eastern Mediterranean University, Mersin 99628, Turkey; 2 Department of Anatomy, School of Medicine, Ankara University, Ankara 06110, Turkey; 3 Department of Dentomaxillofacial Radiology, Faculty of Dentistry, Near East University, Mersin 99138, Turkey; secil.aksoy@neu.edu.tr; 4 Department of Biostatistics, School of Medicine, Eastern Mediterranean University, Mersin 99628, Turkey; ilke.cetin@emu.edu.tr; 5 Department of Dentomaxillofacial Radiology, Faculty of Dentistry, Ankara University, Ankara 06110, Turkey; knorhan@dentistry.ankara.edu.tr; 6 Medical Design Application and Research Center (MEDITAM), Ankara University, Ankara 06110, Turkey; 7 Department of Oral Diagnostics, Faculty of Dentistry, Semmelweis University, 1085 Budapest, Hungary

**Keywords:** nasal concha, maxillary sinus, volumetric assessment, skeletal malocclusion, CBCT

## Abstract

**Background:** This study aimed to evaluate the volumetric characteristics of the inferior and middle nasal conchae and maxillary sinuses in individuals with different skeletal malocclusion classes and cranio-maxillary relationships using cone beam computed tomography (CBCT). **Methods**: A total of 150 adult patients were retrospectively analyzed. CBCT scans were used to obtain volumetric measurements of the right and left inferior nasal conchae (INC), middle nasal conchae (MNC), and maxillary sinuses (MS). Patients were categorized into skeletal Classes I, II, or III based on ANB angles, and into retrognathic, normal, or prognathic groups according to SNA angles. Gender- and age-related differences were also analyzed. Statistical comparisons were performed using appropriate parametric and non-parametric tests. **Results:** Class II individuals exhibited significantly lower conchal volumes compared to Class I and III groups, while MS volumes were highest in Class II, although statistical significance was reached only on the left side. Gender differences were evident, with males presenting greater volumes than females in both the right and left INC and MS; however, significant differences were observed only for the left INC and left MS. A significant age-related decrease in left INC volume was found between the 21–30 and 61+ age groups. No statistically significant correlation was detected between conchal and sinus volumes. **Conclusions:** Skeletal malocclusion patterns, gender, and age significantly influence concha and sinus volumes. These findings emphasize the utility of CBCT-based three-dimensional assessments in enhancing diagnostic accuracy and informing interdisciplinary treatment planning in orthodontics and craniofacial care.

## 1. Introduction

The viscerocranium, which encompasses key anatomical components such as the nasal conchae and nasal cavity, plays an integral role in the development and structural configuration of the craniofacial complex. These components develop in a coordinated and interconnected manner, beginning early in fetal life and continuing through postnatal growth, particularly in conjunction with the paranasal sinuses and maxillofacial bones [1].

Among these, the nasal conchae—bony projections forming the lateral walls of the nasal cavity—are of particular anatomical and functional significance [2,3]. They participate in regulating nasal airflow, humidification, and filtration, and their development is closely linked with adjacent structures, including the maxillary sinuses [1]. The growth of these structures is strongly influenced by respiratory patterns [4].

Alterations in normal breathing, especially chronic mouth breathing induced by conditions such as adenoid hypertrophy, allergic rhinitis, or nasal concha enlargement, may disrupt the balance of orofacial forces. In children, persistent mouth breathing may result in maladaptive tongue posture, reduced muscle tone in the lips and cheeks, and a lowered resting position of the tongue [5]. These conditions may contribute to the development of malocclusions and craniofacial growth disturbances [6].

In addition to their impact on dental occlusion and facial symmetry, altered breathing patterns can influence the pneumatization and volumetric growth of the paranasal sinuses [7]. Adequate nasopharyngeal airflow is essential for the proper aeration and development of these air-filled spaces. It has been reported that individuals with prolonged oral breathing patterns tend to exhibit significantly reduced maxillary sinus volumes when compared to nasal breathers [8].

Skeletal malocclusions, which are typically classified based on the sagittal relationship between the maxilla and mandible, may also correlate with anatomical variations in the nasal cavity and paranasal sinuses [9]. Understanding the volumetric characteristics of nasal conchae and maxillary sinuses has important clinical implications. Differences in these anatomical structures may influence nasal airflow, orthodontic treatment outcomes, and surgical planning in patients with skeletal malocclusion. In particular, volumetric variations can provide additional diagnostic information for orthodontists and maxillofacial surgeons when planning interventions such as rapid maxillary expansion, orthognathic surgery, or the management of airway-related problems. However, no study has been found in the literature regarding the volumetric data of nasal conchae and sinuses across different skeletal malocclusion types.

Therefore, this study aimed to investigate the volumetric characteristics of the maxillary sinuses and nasal conchae—specifically the inferior and middle conchae—in individuals with different skeletal malocclusion classes, utilizing cone beam computed tomography (CBCT). Understanding these anatomical relationships could provide insights into the etiopathogenesis of malocclusion and offer guidance for clinical planning in orthodontics and maxillofacial interventions.

## 2. Materials and Methods

This study adhered to the Declaration of Helsinki guidelines, with ethical approval granted by the Near East University Scientific Research Evaluation Ethics Committee (approval number: YDU 2019/67-785). Written informed consent was obtained from all participants prior to undergoing CBCT imaging.

In this study, the effect size, calculated as Cohen’s f, was found to be 0.258. This effect size was utilized to determine the sample size required to achieve sufficient statistical power. The study was designed with a 95% confidence level and an 80% theoretical power. To meet these criteria, the study was conducted with a total of 150 participants, with at least 50 subjects allocated to each skeletal malocclusion group. A retrospective analysis was conducted of 150 full-head CBCT scans from patients treated at the Near East University, Faculty of Dentistry Department of Dentomaxillofacial Radiology between 2009 and 2017. The study consisted of 86 female and 64 male patients with a mean age of 38.67 ± 16.53 years old. These scans were initially performed for various diagnostic purposes including pre-osteotomy planning, paranasal sinus evaluation, implant planning, impacted teeth surgery and temporomandibular joint disease assessment.

The study included adult participants aged 18 years and older. Exclusion criteria encompassed patients with known hereditary or systemic diseases that could potentially influence the study’s outcomes, as well as those with tumoral lesions in the regions of interest or prior surgical interventions affecting these areas. Additionally, CBCT scans that exhibited artifacts such as scatter caused by metallic dental fillings, or any other conditions that compromised the clarity or diagnostic quality of the images, were also excluded from the study.

The Newtom 3G (Quantitative Radiology s.r.l., Verona, Italy) was used for CBCT imaging. Patients were positioned horizontally with their teeth in natural occlusion, and their head and jaw stabilized; the Frankfurt horizontal plane was maintained perpendicular to the ground. Imaging parameters included isotropic voxels with an axial slice thickness of 0.3 mm, operating at 120 kVp and 3–5 mA within a 12-inch field. All evaluations were carried out on a 21.3-inch flat panel color active matrix TFT medical [(Nio Color 3MP, Barco, Belgium), a resolution of 2048 × 1536 at 76 Hz] screen.

**Figure 1:**
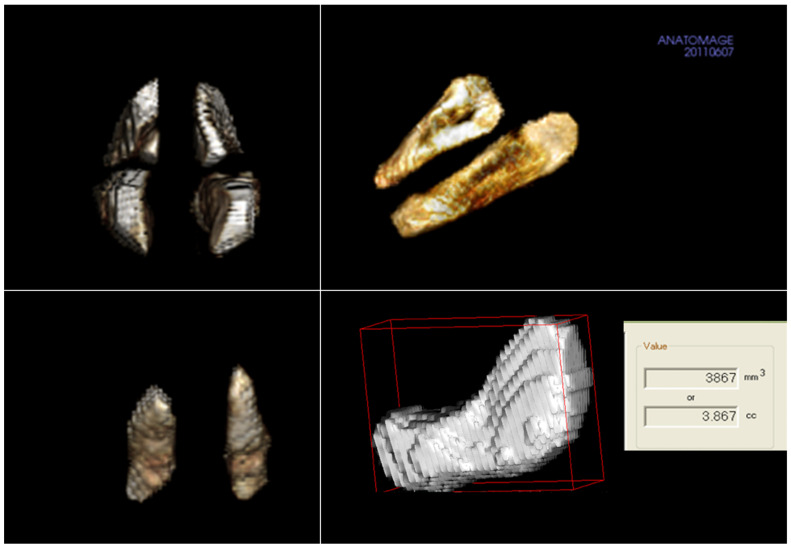
Three-dimensional volume renderings and segmentation of the INC, and MNC obtained from CBCT images. The segmented structures represent the measured volumes of the INC, as part of the volumetric assessment process.

**Figure 2:**
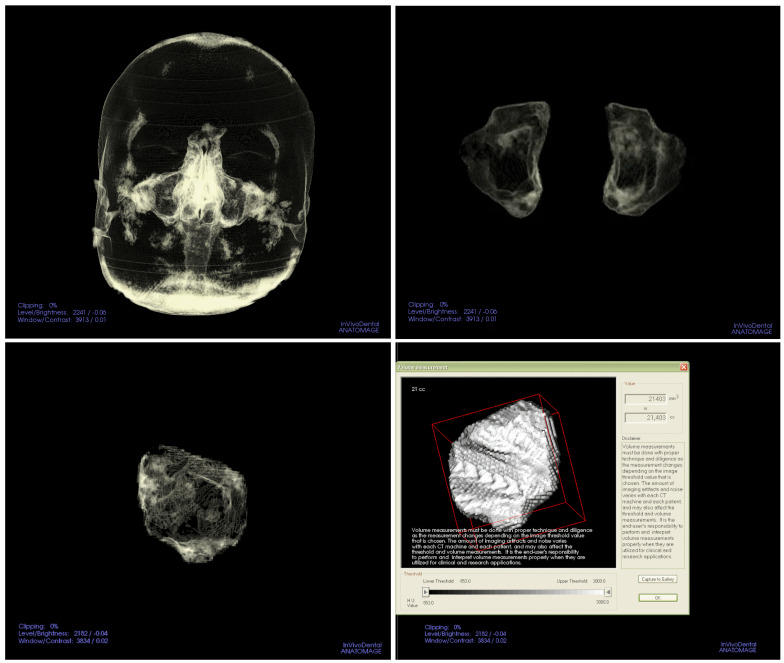
Three-dimensional CBCT image volume renderings and segmentations of the MS. The figure illustrates bilateral maxillary sinus boundaries delineated for volumetric analysis.

Axial images, exported in DICOM format, were imported into the InVivoDental 5.1.2^®^ software (Anatomage Inc., San Jose, CA, USA) for analysis. For volume measurements of the nasal conchae (Figure 1) and maxillary sinuses (Figure 2), we adjusted the “Soft Tissue 1” visualization setting in the “Volume Render” tab. This involved fine-tuning the brightness and opacity to optimize the visibility of the soft tissue structures within the nasal cavity. Each nasal concha and sinus was clearly delineated using the software’s segmentation/cutting tool, adhering to anatomical boundaries to isolate the areas of interest. The “Volume Measurement” tool in the software was utilized to measure the volumetric data. Volume measurements of the maxillary sinus, inferior and middle nasal conchae, and 3D cephalometric analysis were conducted using the InVivoDental program.

Angular measurements were conducted based on cephalometric landmarks identified in this study, which helped assess the spatial orientation of the maxilla and mandible. Specifically, the SNA angle was used to determine the position of the maxilla, the SNB angle to assess the position of the mandible, and the ANB angle to evaluate the maxillomandibular relationship within the midsagittal plane. Subsequently, skeletal classifications were categorized based on the ANB angle values: Class I (0–4°), Class II (>4°), and Class III (<0°). Also to assess the effect of sagittal maxillary position on the measured volumes, patients were classified into three categories based on their SNA angle: retrognathic (SNA < 80°), normal (SNA 80–84°), and prognathic (SNA > 84°).

To assess intra-observer reliability, the same observer re-evaluated a random subset of the images after a 2-week interval. The intra-observer reliability was calculated using the Intraclass Correlation Coefficient (ICC).

For the evaluation of age-related differences, participants were divided into six categories (18–20, 21–30, 31–40, 41–50, 51–60, and 61+ years). These intervals were selected to ensure a balanced distribution across decades and to reflect distinct physiological stages of adulthood. This categorization allowed the detection of potential volumetric changes not only between younger and older adults but also across intermediate decades.

Statistical analyses were performed using SPSS 23 (IBM Corp., Armonk, NY, USA). Data normality was assessed with the Shapiro–Wilk test, guiding the selection of appropriate statistical tests. Depending on the normality of the data, the Independent sample *t*-test and ANOVA were used for normally distributed variables, complemented by the Bonferroni correction. For non-normally distributed variables, the Kruskal–Wallis H test was applied. A significance level of 0.05 was maintained throughout, where *p*-values below 0.05 were considered statistically significant.

The intra-observer reliability analysis yielded an ICC of 0.92, confirming excellent measurement consistency.

## 3. Results

The overall average volumes for both genders were as follows: The average volume for the right inferior nasal conchae was 5.64 cm^3^, and for the left inferior nasal conchae, it was 5.75 cm^3^. The right middle nasal conchae had an average volume of 2.61 cm^3^, while the left middle nasal conchae averaged 2.58 cm^3^. Regarding the maxillary sinuses, the average volume of the right maxillary sinus was 14.30 cm^3^, and the left maxillary sinus was 14.38 cm^3^.

Gender-based differences were observed in the volumetric analysis of nasal conchae and maxillary sinuses. The average volumes for the right and left inferior nasal conchae in males were 5.82 cm^3^ and 6.27 cm^3^ respectively, compared to 5.50 cm^3^ and 5.36 cm^3^ in females. This difference was statistically significant for the left inferior nasal conchae (*p* = 0.001). Also, while the average volumes for the right and left maxillary sinuses in males were 15.51 cm^3^ and 15.82 cm^3^ respectively, females exhibited lower volumes of 13.41 cm^3^ and 13.31 cm^3^ respectively, with the difference in the left maxillary sinus being significant (*p* = 0.007). These findings suggest that there are discernible gender-based differences in the volumetric dimensions of these anatomical regions (Table 1).

Similarly, age-group differences were observed in the volumetric analysis of nasal conchae and maxillary sinuses. Patients were categorized into the six age groups and the average volumes of these structures and their standard deviations were calculated for each group. The findings indicate significant volumetric differences only in the left inferior conchae volume, notably between the 21–30 and 61+ age groups (*p* = 0.013). No significant difference was observed for the other structures between the age groups (Table 2).

The analysis of nasal conchae and maxillary sinus volumes across skeletal malocclusion classes revealed significant differences, especially in the nasal conchae. The inferior and middle nasal conchae displayed notable variations between the malocclusion classes, with Class II generally exhibiting lower volumes both compared to Class I and Class III patients. These differences underscore the impact of skeletal malocclusion on nasal conchae volume. In contrast, differences in maxillary sinus volumes among the classes were less and, in most cases, did not reach statistical significance, suggesting that malocclusion has a more variable impact on these structures (Table 3).

The average volumes of the inferior nasal conchae (INC), middle nasal conchae (MNC), and maxillary sinuses (MS) were compared across SNA groups. While mean values for each structure varied among the different groups, none of the differences reached statistical significance. The right maxillary sinus volume showed the most notable variation, with prognathic individuals demonstrating the highest average volume (15.27 ± 6.78 cm^3^) and the normal group the lowest (12.89 ± 5.98 cm^3^), though this difference was not statistically significant (*p* = 0.119). Similar nonsignificant trends were observed for the inferior and middle nasal conchae (Table 4).

**Figure 3:**
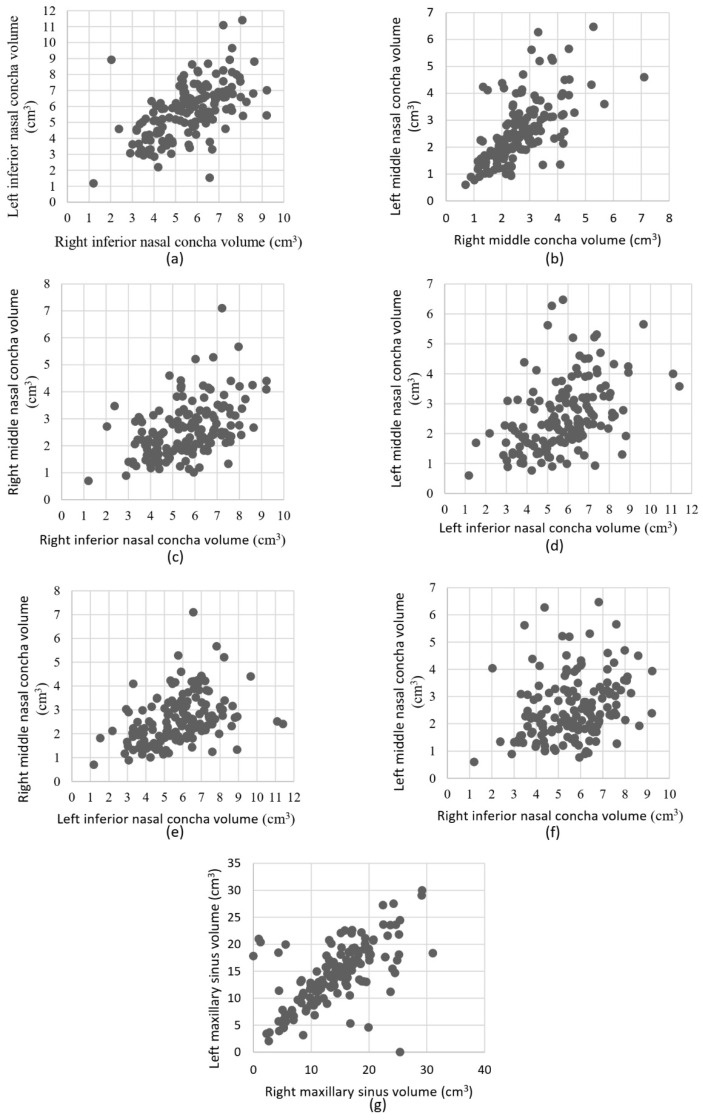
Scatter plots illustrating the correlations between bilateral and inter-structural volumetric measurements of nasal conchae and maxillary sinuses. (**a**) Left vs. right INC volumes (**b**) Left vs. right MNC volumes (**c**) Right INC vs. right MNC volumes (**d**) Left INC vs. left MNC volumes (**e**) Left INC vs. right MNC volumes (**f**) Right INC vs. left MNC volumes (**g**) Left vs. right maxillary sinus (MS) volumes.

Table 5 presents a comprehensive correlation analysis among various nasal and maxillary sinus structures. The Pearson correlation coefficients and their significance levels revealed notable relationships: A significant correlation exists between the right and left inferior nasal conchae, suggesting a symmetrical growth or reduction pattern in nasal structures across the population. The middle nasal conchae (right and left) also show a strong positive correlation with each, which further supports the bilateral symmetry in nasal anatomy. Additionally, the right maxillary sinus volume is strongly correlated with the left maxillary sinus volume, indicating consistent volumetric changes between the two sides (Figure 3). Age showed negative correlations with inferior nasal conchae and maxillary sinus in both sides, suggesting a decrease in volume with increasing age. Middle nasal conchae did not correlate with age. Interestingly, no significant correlations were observed between inferior and middle nasal conchae and the maxillary sinuses.

## 4. Discussion

This study assessed the three-dimensional volumes of the inferior and middle nasal conchae and maxillary sinuses in individuals with different skeletal malocclusion classes using CBCT imaging. The results revealed statistically significant differences in nasal conchal volumes among skeletal classifications, with Class II individuals consistently exhibiting lower nasal conchae volumes. Interestingly, these individuals also demonstrated the highest maxillary sinus volumes, despite the absence of a statistically significant correlation between conchal and sinus volumes.

### 4.1. Gender Differences

Numerous anatomical studies have assessed nasal conchae and maxillary sinus volumes to better understand their structural variability and potential clinical implications. In the present study, although male participants exhibited higher mean volumes in all measured regions, statistically significant gender differences were only observed in the left inferior nasal concha and the left maxillary sinus. A borderline difference was also noted in the right maxillary sinus volume (*p* = 0.055).

These findings are partially consistent with previous literature. Turhan et al. [10] reported significantly larger conchal volumes in male subjects, while Emirzeoğlu et al. [11] observed gender-related differences only in the MNC but not in the INC. Similarly, Shetty et al. [12] found no statistically significant gender-based differences in total INC volume. Pediatric research by Ertekin et al. [13] demonstrated an increase in conchal volumes in both sexes during growth, with gender differences becoming significant only after the completion of craniofacial development. Since our study comprised only adults, age-dependent developmental differences may not have been captured.

Additionally, a study evaluating inferior nasal concha volumes in patients with obstructive sleep apnea (OSA) found significantly higher INC volumes in OSA patients compared to healthy controls (right INC: 5.57 ± 2.14 cm^3^ vs. 4.34 ± 1.77 cm^3^; left INC: 5.12 ± 2.04 cm^3^ vs. 3.90 ± 1.07 cm^3^) [14]. Interestingly, the conchal volumes observed in the present study closely resemble those of the OSA group, despite the exclusion of individuals with diagnosed respiratory disorders. This similarity may suggest underlying anatomical predispositions or subclinical variability. Moreover, the higher volumetric values compared to earlier stereological studies may be attributed to differences in imaging modalities (CBCT vs. CT), segmentation protocols, or population-based anatomical variation.

Gender-related differences in maxillary sinus volumes have also been widely studied, with most findings indicating larger MS volumes in males [9,15]. In line with these reports, male participants in the present study showed significantly greater left MS volumes, and a borderline significant difference on the right side. Aktuna Belgin et al., Dinç and İçöz, and Oruç et al. similarly reported significantly larger MS volumes in males [16,17,18]. These consistent findings suggest a robust biological basis for sexual dimorphism in maxillary sinus dimensions, possibly linked to overall differences in craniofacial size and morphology.

### 4.2. Age-Related Differences

Several studies have highlighted the influence of age on the volumetric development of nasal and paranasal structures. Prior research has demonstrated that nasal cavity and paranasal sinus volumes tend to increase during childhood and adolescence, reaching a peak in early adulthood, followed by a gradual volumetric decrease with advancing age. For instance, Velasco-Torres et al. and Ariji et al. reported that maxillary sinus volumes peak around the third decade of life, after which sinus pneumatization appears to regress slightly [19,20].

In a stereological study by Ertekin et al., nasal conchae volumes were shown to increase progressively until age 15, after which significant gender-based differences became apparent [13]. Similarly, Turhan et al. compared nasal structure volumes in adults and children using CBCT and found statistically significant differences in all anatomical regions [10]. Specifically, adults had consistently higher volumes than children across both the inferior and middle nasal conchae as well as the nasal septum. Since our study exclusively included adult participants, the age-related volumetric increases reported in pediatric populations may not be directly observable in our cohort. Nevertheless, the subtle volume reduction in older age groups supports previous evidence of anatomical remodeling and possible mucosal involution in the nasal cavity over time.

Collectively, these observations align with known craniofacial growth trajectories and emphasize the relevance of age when interpreting volumetric data of the nasal and paranasal structures.

### 4.3. Skeletal Classification Differences

This study uniquely contributes to the literature by evaluating the volumes of nasal conchae and maxillary sinuses across different skeletal malocclusion classes and cranio-maxillary relationships. In our results, Class II individuals exhibited significantly reduced nasal concha volumes alongside increased maxillary sinus volumes. This dual alteration may point to complex compensatory mechanisms in craniofacial development.

Nearly consistent with our findings, Dinç and İçöz observed that skeletal pattern did not influence MSV in terms of ANB or SNA-based classifications [17]. The present study only found significant differences in the Class II group, showing higher MS volume on the left side. Similarly, Oruç et al. found no significant difference in MSV among malocclusion classes [18]. In contrast, Shrestha et al. reported that individuals with skeletal Class II malocclusion had significantly larger MSV than those with Class III malocclusion [21]. Abate et al., in their comprehensive 3D analysis, found that although MSV and surface area did not differ significantly between ANB groups, patients with retrusive (SNA < 80°) or protrusive (SNA > 84°) maxillae exhibited greater MSV and linear dimensions compared to those with a normal SNA [22]. Although our findings were similar to those of previous studies, and the normal group exhibited lower MSV than the others, this result did not reach statistical significance.

In light of these comparisons, present study offers a broader anatomical perspective by incorporating the nasal conchae into the analysis. The inclusion of volumetric nasal concha assessment provides novel insights into how different skeletal patterns may influence not only sinus development but also adjacent nasal structures. Given the lack of prior studies exploring this relationship, the present study results highlight the need to broaden volumetric evaluations beyond the maxillary sinuses when investigating craniofacial variations in orthodontic populations.

Although maxillary sinus volumes have been extensively studied in relation to skeletal malocclusions, the inferior and middle nasal conchae have not been evaluated in this context. Their inclusion provides clinically relevant insights, as smaller bony conchae may indicate relatively wider nasal passages, whereas larger ones may suggest narrower spaces. Such distinctions can influence orthodontic treatment planning, rapid maxillary expansion, and orthognathic surgery.

The potential correlation between nasal conchae and maxillary sinus volumes has been a subject of ongoing investigation. However, in the present study, no statistically significant correlation was observed between the volumetric measurements of the inferior or middle nasal conchae and the maxillary sinuses. This finding is consistent with the results of multiple studies. For instance, Tassoker et al. reported no significant correlation between MSV and anatomical variations such as nasal septal deviation (NSD) and concha bullosa (CB) [23]. In contrast, Al-Rawi et al. found that while CB was associated with increased MSV, NSD showed no significant impact on sinus volume, suggesting that the presence of certain anatomical variations may not consistently influence sinus pneumatization [24]. Kapusuz Gencer et al. further examined the influence of NSD and found that contralateral maxillary sinus volumes were significantly larger in cases of severe deviation [25]. However, no correlation was found between the degree of deviation and overall MSV in milder cases, indicating that only severe anatomical alterations may induce volumetric adaptations in adjacent structures.

Taken together, these findings support the view that although nasal and paranasal structures are anatomically and functionally interconnected, their volumetric development may follow independent pathways or be influenced by additional factors such as local airflow dynamics, chronic mucosal conditions, or genetic predispositions. Further studies incorporating functional assessments and standardized segmentation protocols are needed to elucidate the interplay between nasal cavity morphology and paranasal sinus development.

Although volumetric evaluation of nasal conchae and maxillary sinuses is not currently a standard component of diagnostic protocols, our findings indicate that such analyses can provide valuable supplementary information in selected clinical scenarios. Since our study assessed the osseous structure of the conchae, smaller bony volumes may actually reflect relatively wider airway passages, whereas larger conchal volumes may correspond to narrower spaces. This nuance is important when interpreting the clinical relevance of volumetric measurements. In orthodontic patients with skeletal Class III malocclusion, in cases with suspected airway obstruction such as obstructive sleep apnea, or in preoperative planning for orthognathic and sinus surgery, these assessments may contribute to a more comprehensive understanding of craniofacial and airway morphology.

These results have important clinical implications, particularly in interdisciplinary planning involving airway management, orthodontic expansion, and orthognathic surgery. Furthermore, volumetric assessment via CBCT offers a practical, reliable method for non-invasive evaluation of airway-related structures.

Our findings further confirm that nasal conchae volumes are influenced by gender and age, independent of skeletal classification. The consistent trend of higher volumes in male individuals and volume reduction in older age groups aligns with previous anatomical studies and enhances our understanding of upper airway morphology in orthodontic populations.

However, this study has certain limitations. First, the retrospective design restricts control over confounding factors such as breathing patterns, nasal pathologies, or previous treatments that might influence volumetric outcomes. Second, the study population consisted largely of patients seeking dental or orthodontic care, which may introduce sampling bias toward individuals with craniofacial anomalies. Additionally, functional assessments of airway resistance or airflow, such as rhinomanometry or acoustic rhinometry, were not included, which limits the correlation between anatomical volume and functional outcomes.

Future research should incorporate longitudinal follow-up, a broader population base, and integrate functional assessments to explore how anatomical variation translates into clinical symptoms or treatment outcomes. Combining volumetric analysis with airflow modeling or respiratory function testing could further improve interdisciplinary care in orthodontics, otolaryngology, and sleep medicine.

## 5. Conclusions

This study highlights significant volumetric differences in nasal conchae and maxillary sinuses across skeletal malocclusion types. Class II individuals had smaller conchae and larger sinus volumes. Gender and age were influential, with males showing higher volumes and older individuals showing reductions. These findings underline the potential clinical relevance of CBCT-based volumetric assessment. Although not part of standard diagnostic protocols, such measurements may provide supplementary information in selected scenarios, such as evaluation of suspected airway obstruction, or preoperative assessment before orthognathic and sinus surgery. Incorporating these volumetric data into clinical decision-making could therefore enhance both diagnostic accuracy and treatment outcomes.

## Figures and Tables

**Table 1:** Comparative analysis of nasal conchae and maxillary sinus volumes by gender.

Anatomical Structure		Gender	Average Volume ± SD (cm^3^)	*p* Value
Inferior nasal conchae (INC)	Right	Female	5.50 ± 1.52	0.201
		Male	5.82 ± 1.51	
	Left	Female	5.36 ± 1.43	0.001 *
		Male	6.27 ± 1.96	
Middle nasal conchae (MNC)	Right	Female	2.57 ± 1.04	0.540
		Male	2.67 ± 1.02	
	Left	Female	2.46 ± 1.09	0.156
		Male	2.75 ± 1.27	
Maxillary sinus (MS)	Right	Female	13.41 ± 5.28	0.055
		Male	15.51 ± 7.37	
	Left	Female	13.31 ± 5.35	0.007 *
		Male	15.82 ± 5.94	

* Statistical significance at *p* < 0.05.

**Table 2:** Comparison of nasal conchae and maxillary sinus volumes in different age groups.

Anatomical Structure		Age Groups	*n*	Average Volume ± SD (cm^3^)	*p* Value
INC	Right	18–20	17	6.00 ± 1.36	0.100
		21–30	46	5.78 ± 1.36	
		31–40	22	5.48 ± 1.61	
		41–50	20	5.87 ± 1.49	
		51–60	25	5.78 ± 1.50	
		61+	20	4.75 ± 1.78	
	Left	18–20	17	5.90 ± 1.21	0.037 * 21–29 vs. 61+ *p* = 0.013 *
		21–30	46	6.13 ± 1.74	
		31–40	22	5.61 ± 1.40	
		41–50	20	5.81 ± 1.58	
		51–60	25	5.80 ± 1.73	
		61+	20	4.75 ± 2.28	
MNC	Right	18–20	17	2.60 ± 0.93	0.838
		21–30	46	2.69 ± 1.23	
		31–40	22	2.66 ± 0.90	
		41–50	20	2.45 ± 0.83	
		51–60	25	2.73 ± 0.98	
		61+	20	2.38 ± 1.01	
	Left	18–20	17	2.74 ± 1.26	0.693
		21–30	46	2.55 ± 1.15	
		31–40	22	2.78 ± 1.14	
		41–50	20	2.45 ± 1.01	
		51–60	25	2.72 ± 1.41	
		61+	20	2.25 ± 1.09	
MS	Right	18–20	17	16.54 ± 7.35	0.184
		21–30	46	15.49 ± 5.70	
		31–40	22	13.41 ± 7.12	
		41–50	20	14.50 ± 7.03	
		51–60	25	12.68 ± 6.39	
		61+	20	12.48 ± 4.21	
	Left	18–20	17	15.62 ± 6.11	0.156
		21–30	46	15.58 ± 5.47	
		31–40	22	12.67 ± 6.17	
		41–50	20	15.29 ± 6.35	
		51–60	25	13.08 ± 5.24	
		61+	20	13.18 ± 4.95	

* Statistical significance at *p* < 0.05.

**Table 3:** Volumetric differences in nasal conchae and maxillary sinuses across different skeletal malocclusion classes.

Anatomical Structure		Malocclusion Class	Average Volume ± SD (cm^3^)	*p* Value	Pairwise Comparison
INC	Right	Class I	5.91 ± 1.46	<0.0005 *	CI vs. CII *p* = 0.003 *
		Class II	4.95 ± 1.44		CI vs. CIII *p* = 0.899
		Class III	6.04 ± 1.45		CII vs. CIII *p* = 0.001 *
	Left	Class I	6.03 ± 1.38	<0.0005 *	CI vs. CII *p* < 0.0005 *
		Class II	4.74 ± 1.62		CI vs. CIII *p* = 0.370
		Class III	6.46 ± 1.71		CII vs. CIII *p* < 0.0005 *
MNC	Right	Class I	3.01 ± 0.89	<0.0005 *	CI vs. CII *p* < 0.0005 *
		Class II	1.99 ± 1.04		CI vs. CIII *p* = 0.226
		Class III	2.84 ± 0.84		CII vs. CIII *p* < 0.0005 *
	Left	Class I	2.96 ± 1.08	<0.0005 *	CI vs. CII *p* < 0.0005 *
		Class II	1.73 ± 0.79		CI vs. CIII *p* = 0.705
		Class III	3.06 ± 1.14		CII vs. CIII *p* < 0.0005 *
MS	Right	Class I	14.31 ± 5.68	0.662	-
		Class II	14.83 ± 5.58		-
		Class III	13.77 ± 7.59		-
	Left	Class I	14.62 ± 5.20	0.011 *	CI vs. CII *p* = 0.458
		Class II	15.95 ± 4.37		CI vs. CIII *p* = 0.165
		Class III	12.58 ± 6.9		CII vs. CIII *p* = 0.008 *

* Statistical significance at *p* < 0.05.

**Table 4:** Volumetric differences in nasal conchae and maxillary sinuses among SNA groups.

Anatomical Structure		SNA Based Classification	Average Volume ± SD (cm^3^)	*p* Value
INC	Right	Retrognathic	5.78 ± 1.59	0.611
		Normal	5.52 ± 1.59	
		Prognathic	5.53 ± 1.27	
	Left	Retrognathic	6.01 ± 1.78	0.260
		Normal	5.61 ± 1.85	
		Prognathic	5.46 ± 1.36	
MNC	Right	Retrognathic	2.77 ± 1.09	0.224
		Normal	2.44 ± 0.88	
		Prognathic	2.58 ± 1.10	
	Left	Retrognathic	2.72 ± 1.13	0.435
		Normal	2.53 ± 1.20	
		Prognathic	2.41 ± 1.20	
MS	Right	Retrognathic	15.00 ± 6.22	0.119
		Normal	12.89 ± 5.98	
		Prognathic	15.27 ± 6.78	
	Left	Retrognathic	14.93 ± 5.36	0.243
		Normal	13.33 ± 5.30	
		Prognathic	15.04 ± 6.82	

**Table 5:** The correlation between nasal conchae and maxillary sinus volume with age.

		Right INC	Left INC	Right MNC	Left MNC	Right MS	Left MS	Age
Right INC	Pearson correlation	1	0.573 *	0.443 *	0.271 *	0.148	0.060	−0.182 *
	Sig. (2-tailed)		0.000 *	0.000 *	0.001 *	0.070	0.466	0.026 *
Left INC	Pearson correlation	0.573 *	1	0.389 *	0.448 *	0.132	0.045	−0.199 *
	Sig. (2-tailed)	0.000 *		0.000 *	0.000 *	0.107	0.587	0.015 *
Right MNC	Pearson correlation	0.443 *	0.389 *	1	0.630 *	0.135	0.053	−0.068
	Sig. (2-tailed)	0.000 *	0.000 *		0.000 *	0.099	0.520	0.406
Left MNC	Pearson correlation	0.271 *	0.448 *	0.630 *	1	−0.009	−0.020	−0.058
	Sig. (2-tailed)	0.001 *	0.000 *	0.000 *		0.911	0.807	0.480
Right MS	Pearson correlation	0.148	0.132	0.135	−0.009	1	0.620 *	−0.222 *
	Sig. (2-tailed)	0.070	0.107	0.099	0.911		0.000 *	0.006 *
Left MS	Pearson correlation	0.060	0.045	0.053	−0.020	0.620 *	1	−0.179 *
	Sig. (2-tailed)	0.466	0.587	0.520	0.807	0.000 *		0.029 *

* Statistical significance at *p* < 0.05.

## Data Availability

The data presented in this study are available on request from the corresponding author due to ethical reasons.
